# Data mining–based analysis to explore the application of an animal model of diabetic gastroparesis

**DOI:** 10.3389/fendo.2025.1612473

**Published:** 2025-07-09

**Authors:** Hui Xu, Fu-rui Miao, Yu-jun He, Yu-shan Fan

**Affiliations:** College of Acupuncture-moxibustion and Tuina, Guangxi University of Chinese Medicine, Nanning, China

**Keywords:** diabetic gastroparesis, animal model, data mining, application analysis, detection indexes

## Abstract

**Objective:**

This review aims to study the characteristics of animal models of diabetic gastroparesis, provide a reference for the standardization of model preparation, and offer a better experimental basis for researching its pathogenesis and diagnosis-treatment strategies.

**Methods:**

By searching databases including PubMed, Web of Science, China Knowledge Network, Wanfang Data Knowledge Service Platform, and China Science and Technology Journal Database, we obtained literature on diabetic gastroparesis animal experiments from 2000 to 2024. We assessed the literature for the risk of bias using the Systematic Review Center for Laboratory Animal Experimentation tool. We summarized the animal species, sex, modeling methods, modeling criteria, detection indexes, etc.; established a database using Excel software; and applied SPSS Modeler 18.0 and Cytoscape 3.7.2 to analyze the characteristics of diabetic gastroparesis animal models.

**Results:**

A total of 211 articles were included. It was found that Sprague–Dawley rats were the primary animal model, with male rats predominantly used in modeling. Modeling methods primarily included a one-time injection of streptozotocin (60–65 mg/kg) to induce type 1 diabetic gastroparesis or a one-time injection of streptozotocin (40–55 mg/kg) combined with a high-sugar and high-fat irregular diet to induce type 2 diabetic gastroparesis. Most studies set the modeling period as 8 weeks after drug administration. Blood glucose, general condition, and gastric emptying rate were commonly used as modeling criteria, and domperidone served as the positive control drug. Main detection indexes included blood glucose, general condition, gastrointestinal function dynamics, histopathological analysis, immunohistochemistry, Western blotting, etc.

**Conclusion:**

There is no recognized modeling method and evaluation standard for diabetic gastroparesis animal models. On the basis of the results of data analysis, it is recommended to use a one-time injection of streptozotocin (60–65 mg/kg) to induce type 1 diabetic gastroparesis animal model or a one-time injection of streptozotocin (40–55 mg/kg) combined with irregular feeding of high-sugar and high-fat feed to induce type 2 diabetic gastroparesis animal model. Blood glucose, general condition, and gastric emptying rate were used to judge the models.

## 1 Introduction

Diabetic gastroparesis (DGP) is a common and life-threatening complication of diabetes mellitus, which is characterized by delayed gastric emptying and clinical manifestations of gastrointestinal symptoms such as early satiety, bloating, nausea, and vomiting (1). Gastroparesis affects 8.3% of type 1 diabetes mellitus patients and 12.5% of type 2 diabetes mellitus patients, respectively, seriously affecting their quality of life and increasing their economic burden (2). Existing pharmacological treatments are mainly aimed at symptomatic relief, with limited improvement in the pathology of gastric dyskinesia, and the search for an effective, safe, stable, and reliable treatment is an urgent topic for current research (3).

**Figure 1:**
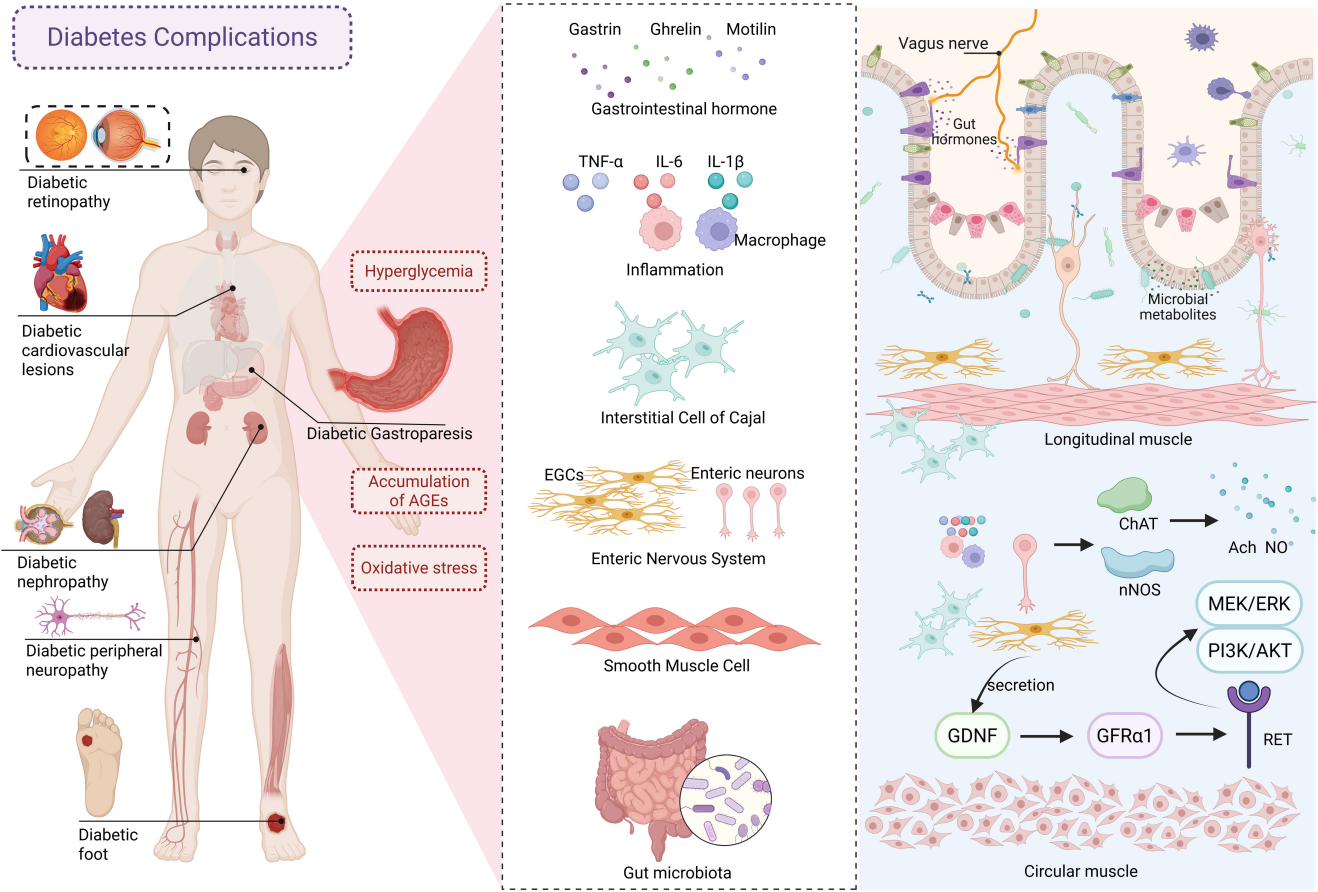
The pathogenesis of diabetic gastroparesis. AGEs, advanced glycation end products; EGCs, enteric glial cells; GDNF, glial cell–derived neurotrophie factor; GFRα1, glial cell–derived neurotrophic factor receptor alpha 1.

Compared with the mechanisms of insulin deficiency and insulin resistance in diabetes, the pathogenesis of DGP focuses more on gastrointestinal neuro-muscular dysfunction, involving autonomic neuropathy, enteric nervous system (ENS) abnormalities, gastrointestinal smooth muscle dysfunction, gastrointestinal hormone disorders, and inflammation. These factors collectively lead to gastrointestinal motor dysfunction and subsequent delayed gastric emptying (4). See **Figure 1** for details. Under long-term hyperglycemia, the accumulation of advanced glycation end products (AGEs) and oxidative stress promotes each other. Gastrointestinal hormones, as key signaling molecules regulating gastrointestinal motility, participate in the pathophysiological processes of DGP by acting on gastric smooth muscle cells, neurotransmitter systems, etc. Among them, motilin can promote gastric antrum contraction, coordinate the migrating motor complex, and induce gastric emptying. Ghrelin and its receptor may regulate the rhythm of gastrointestinal motility by acting on interstitial cells of Cajal (ICC) in the mouse’s small intestine. Current studies have found that DGP patients exhibit disordered gastrointestinal hormone levels, accompanied by increased reactive oxygen species (ROS) activity and malondialdehyde (MDA) content. As the pacemaker cells of the stomach, damage and loss of ICC lead to gastrointestinal motility disorders (5). Chronic hyperglycemia affects the structure and function of intestinal neurons, resulting in decreased viability of enteric neurons and enteric glial cells (EGCs), and induces cellular damage and apoptosis through oxidative stress and inflammatory responses (6, 7). Studies have confirmed the presence of enteric neurons and EGC damage in the gastric antrum of DGP rats (8). Gastrointestinal smooth muscle, as the executive unit of motility, produces rhythmic contraction and relaxation under the dual regulation of ICC slow-wave potentials and ENS signals. Hyperglycemia can activate related signaling pathways through oxidative stress, inducing extracellular matrix remodeling in the smooth muscle of DGP rats (9). It is noteworthy that the role of intestinal microecological imbalance in DGP has been gradually revealed in recent years, and researchers have improved the symptoms of DGP by regulating the composition of intestinal flora (10). Given that human studies are limited by ethical and sampling difficulties, animal models have become an irreplaceable tool for studying the pathogenesis and therapeutic mechanisms of DGP. Therefore, the establishment of an animal model of DGP that meets the characteristics of clinical disease and has good reproducibility is a top priority.

In recent years, many scholars have conducted a large number of experiments and studies on DGP animal models, creating a variety of feasible modeling methods, but there is no uniform standard animal model. Some reviews have summarized DGP animal models, but they lacked the support of objective data, while the dosage of modeling drugs, the modeling period, and modeling standards were not described (11, 12). Data mining refers to the process of extracting potentially useful information and knowledge hidden in large amounts of incomplete, noisy, vague, and random real-world application data (13). Currently, many studies (14–16) are leveraging data mining techniques to parse patterns latent in complex medical data, opening up entirely new paths for disease prevention, diagnosis, and treatment, as well as for the discovery of medical knowledge. Therefore, this paper summarizes the literature on DGP animal models using data mining and analyzes the animal species, sex, modeling methods, drug dosages, modeling cycles, and modeling criteria to explore the current research status and shortcomings of DGP animal models, to provide a reference basis for establishing a standardized DGP animal model.

## 2 Data and methods

### 2.1 Data sources

Advanced searches were conducted in PubMed, Web of Science, China National Knowledge Infrastructure, Wanfang Data Knowledge Service Platform, and China Science and Technology Journal Database. A comprehensive search for articles with titles or abstracts including the terms “diabetic gastroparesis” was performed, with the search period ranging from the database establishment to 31 December 2024.

### 2.2 Inclusion criteria

a. All the experimental literature dealing with the DGP model and successful modeling. b. Complete information about the model (including animal species, modeling methods, and modeling criteria) is well-documented. c. Full articles are accessible, and the language type is not limited. d. The experiments were approved by the Animal Ethics Committee.

### 2.3 Exclusion criteria

a. Articles with unknown modeling methods and standards are excluded. b. Literature with unknown animal models or literature involving other diabetic complications is excluded. c. Reviews, scientific research results, theoretical discussions, master’s and doctoral dissertations, conference papers, and clinical observations are excluded. d. Duplicated literature in Chinese and English is excluded. e. Simple *in vitro* cellular experiments are excluded.

### 2.4 Database establishment

The retrieved literature was imported into the literature manager, NoteExpress. After removing duplicate literature using the software, two researchers independently conducted a preliminary screening and re-screening of the literature according to inclusion and exclusion criteria and verified the screening results to ensure the accuracy of the included literature. Information such as animal species, gender, modeling methods, modeling criteria, and test indicators from the included literature was input into Excel 2021 to establish a database of DGP animal models.

### 2.5 Literature quality evaluation method

Based on the 10 items and six types of bias covered by the SYRCLE (Systematic Review Center for Laboratory Animal Experimentation) tool, the methodological quality of the included studies was evaluated. Each item was assessed as “low risk,” “unclear risk,” or “high risk.” A study was rated as “low risk” if it used reasonable methods to reduce bias, “unclear risk” if incomplete information made bias assessment impossible, and “high risk” if there were behaviors potentially increasing bias risk.

### 2.6 Statistical analysis

Microsoft Excel 2019 was used for statistical analysis of the data. SPSS Modeler 18.0 was utilized to conduct association rule analysis on model establishment criteria using the Apriori algorithm. Support was defined as the probability of co-occurrence of antecedents and consequents, whereas confidence represented the probability of consequents occurring when antecedents were present. The optimal minimum support and confidence thresholds were determined through multiple trials. Cytoscape 3.7.2 was used to visualize and upgrade the network diagram of association rule analysis results.

## 3 Results

### 3.1 Results of data inclusion

**Figure 2:**
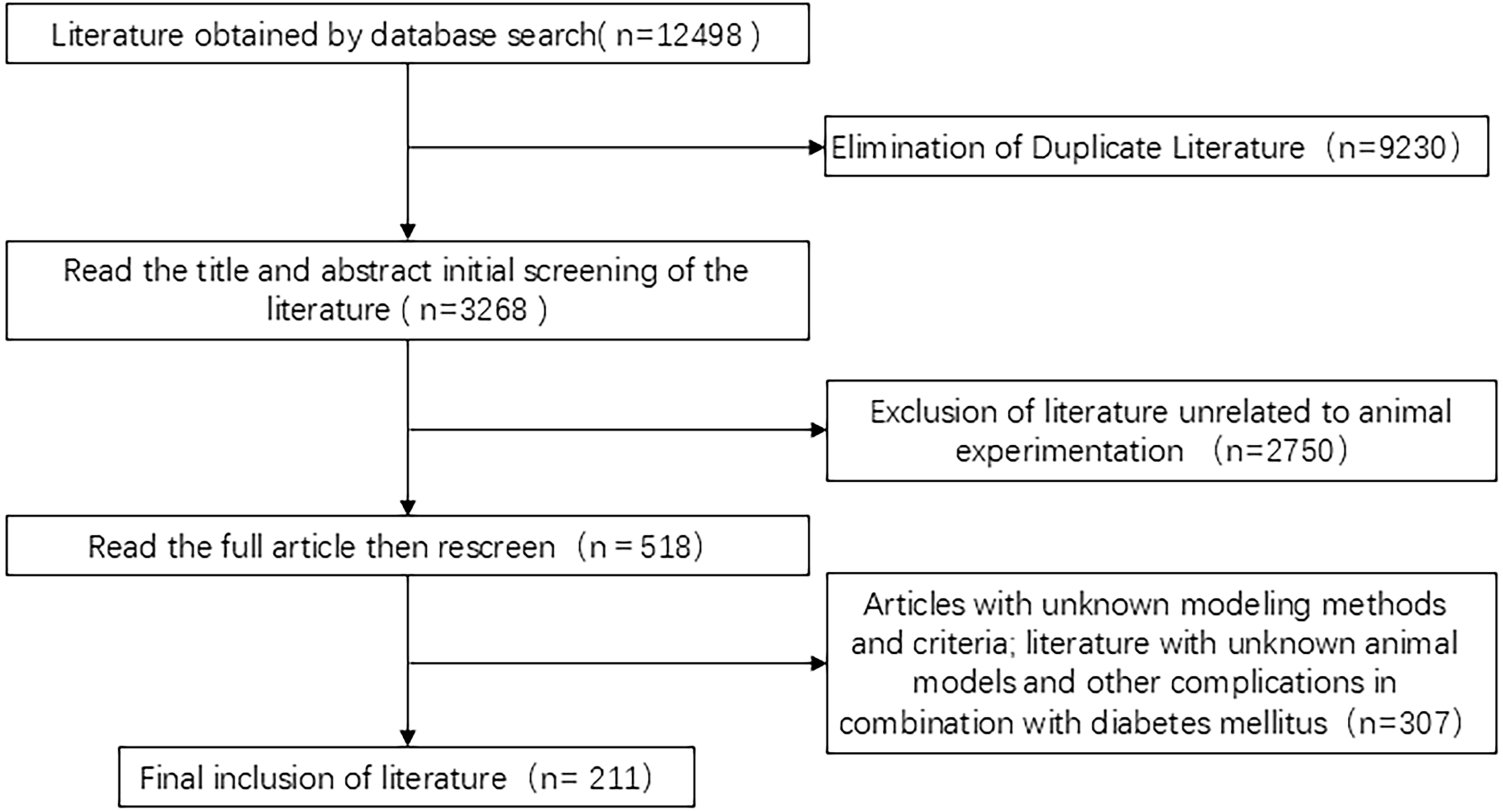
Screening process.

A total of 12,498 related citations were identified. First, 9,230 duplicates were excluded, followed by 2,750 documents excluded after preliminary screening of titles and abstracts. We then obtained 518 articles related to DGP animal experiments and finally included 211 studies after a detailed full-text review and exclusion of those with incomplete model-related information, as shown in **Figure 2**.

### 3.2 Quality of the included studies

The SYRCLE’s risk of bias tool is currently the only instrument specifically designed to evaluate the internal validity of animal experiments (17). Among the 10 items, “selective outcome reporting” was evaluated as “low risk” for 100% of the included studies, as all expected outcomes were reported. “Allocation concealment,” “blinding of experimentalists,” “random outcome assessment,” and “blinding of outcome assessors” had 100% “uncertain risk” due to the absence of explicit information in the literature. In terms of “sequence generation allocation,” 68 studies used random number tables for grouping, rated as “low risk,” whereas the remaining 143 studies only mentioned “random” without specifying the method, classified as “uncertain risk.” For “baseline characteristics,” 42 studies reported detailed baseline data of experimental animals in tables or text, evaluated as “low risk.” Regarding “random housing,” 135 studies specified that animals were kept in identical environments (e.g., temperature, humidity, light cycle, feeding, and watering conditions) and were rated as “low risk.” “Incomplete outcome data” was “high risk” for 60 studies that did not explain or address missing data. For “other biases,” three studies were rated “high risk” due to small sample sizes, which may introduce bias; see **Table 1** for details.

**Table 1:** Risk of bias summary.

Type of bias	Entry	Low risk	Uncertain risk	High risk
Selective bias	Sequence generation	68	143	0
	Baseline characteristics	42	169	0
	Allocation concealment	0	211	0
Implementation bias	Random housing	135	76	0
	Blinding of experimentalists	0	211	0
Measurement bias	Random outcome assessment.	0	211	0
	Blinding of outcome assessors	0	211	0
Lost visit bias	Incomplete outcome data	151	0	60
Reporting bias	Selective outcome reporting	211	0	0
Other bias	Other sources of bias	208	0	3

### 3.3 Animal species

**Figure 3:**
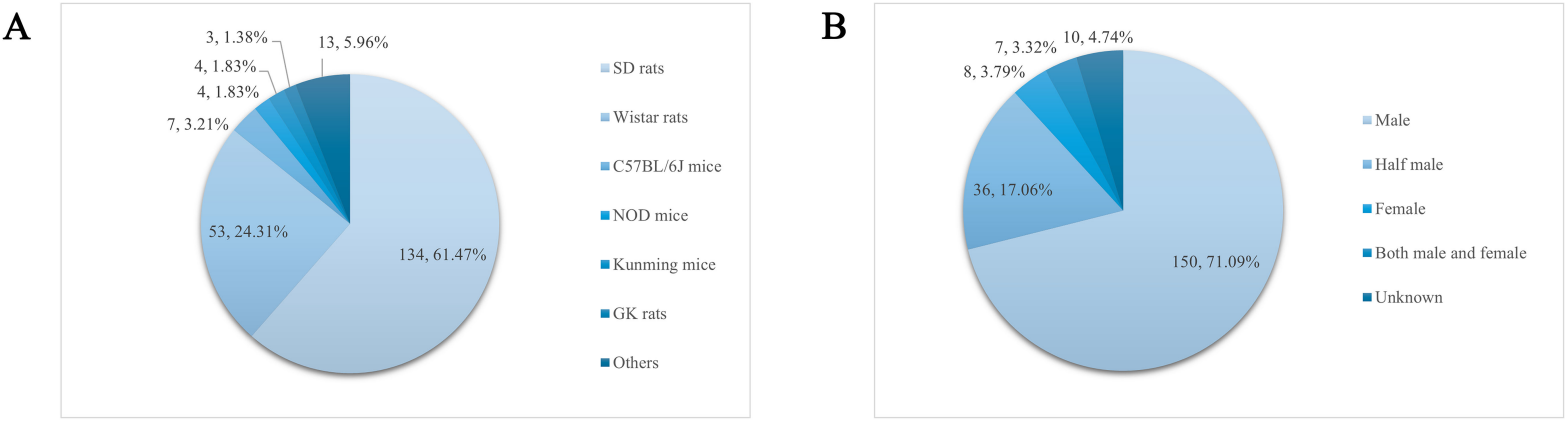
**(A)** Strains of DGP animals and frequency. **(B)** Sex and frequency of DGP animals.

The experimental animals in the recorded 211 papers were categorized and counted, and a total of 13 strains appeared with a cumulative frequency of 218 times. From the statistical results, the animals used to prepare the DGP model were rats, mice, guinea pigs, rabbits, dogs, etc., of which the most used for modeling were Sprague–Dawley (SD) rats (134, 61.47%), and the rest, in order of prevalence (those accounting for more than 1% of the total number of animals were), were Wistar rats (53, 24.31%), C57BL/6J mice (7, 3.21%), Non Obese Diabetes (NOD) mice (4, 1.83%), Kunming mice (4, 1.83%), and Goto-Kakizaki (GK) rats (3, 1.38%), as shown in **Figure 3A**.

### 3.4 Sex of animals

The sex of experimental animals was statistically analyzed in 211 articles entered into the literature, of which 10 articles did not record the sex of experimental animals and were classified as “unknown.” In the remaining 201 articles, the most used animals for modeling were males (150, 71.09%), followed by half males and half females (36, 17.06%), females (8, 3.79%), and both male and female (7, 3.32%), as shown in **Figure 3B**.

### 3.5 Methods of modeling

The modeling methods recorded in the 211 articles were summarized and grouped into three main categories: chemically induced, spontaneous, and composite. Among them, chemically induced (199, 94.31%) was the main modeling method. Among chemically induced, intraperitoneal streptozotocin (STZ) combined with dietary induction (107, 50.71%) was used most frequently, followed by chemical induction: STZ (71, 33.65%), as detailed in **Table 2**.

**Table 2:** DGP modeling methods and their frequencies.

Category	Specific classification	Frequency	Proportions (%)
Chemically induced	Chemically induced: STZ	71	33.65
	Chemically induced: STZ + dietary induction	107	50.71
	Chemically induced: STZ + prepared rhizome of rehmannia aqueous	4	1.90
	Chemically induced: STZ + dietary induction+ prepared rhizome of rehmannia aqueous	1	0.47
	Chemically induced: alloxan	5	2.37
	Chemically induced: alloxan + prepared rhizome of rehmannia aqueous	9	4.27
	Chemically induced: alloxan + STZ	1	0.47
	Chemically induced: alloxan + dietary induction	1	0.47
Spontaneous	Spontaneous	4	1.90
	Spontaneous + dietary induction	7	3.32
Mixed	Chemically induced: STZ + spontaneous/dietary induction	1	0.47

### 3.6 Dosage of chemically induced substances

**Figure 4:**
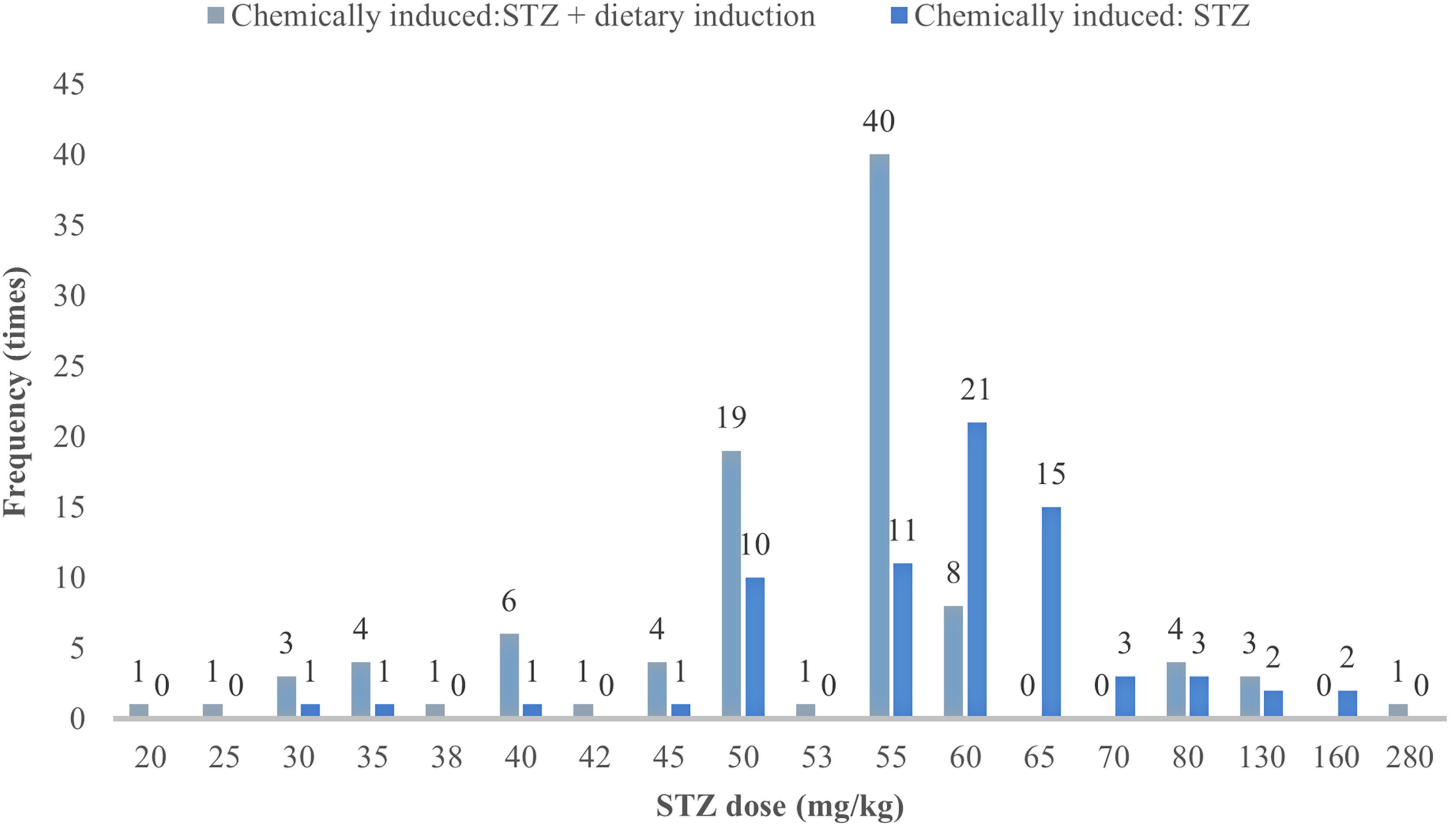
One-time injection of STZ dose and its frequency in different induction methods.

In animal studies of DGP, STZ alone is mostly used to induce type 1 DGP, whereas dietary induction combined with low-dose STZ is mostly used for type 2 DGP (18). The study analyzed 185 publications on the use of STZ in the preparation of animal models of DGP (87.7% of the total number of publications included), and STZ was divided into three groups according to dose: low dose (<40 mg/kg), medium dose (40–55 mg/kg), and high dose (>60 mg/kg). Because 70 mg/kg in the high dose was prone to cause death in rats (19). The high dose was further subdivided into 60–65 mg/kg, and 70 mg/kg. The results showed that the modeling with STZ alone was taken as a one-time intraperitoneal injection, and the dose of 60–65 mg/kg was used most frequently (36, 19.46%), whereas the one-time injection dose of 40–55 mg/kg was used most frequently for STZ in combination with diet-induced gastroparesis in type 2 diabetes accounted for the largest proportion (71, 38.38%), and multiple injections were only reported in 10 papers; see **Table 3** and **Figure 4** for details.

**Table 3:** Different injection volumes and frequencies of STZ.

Modeling method	Injection method	Dose (mg/kg)	Frequency	Proportions (%)
Chemically induced: STZ	One-time injection	<40	2	1.08
		40–55	22	11.89
		60–65	36	19.46
		>70	10	5.41
Chemically induced: STZ + dietary induction	One-time injection	<40	10	5.41
		40–55	71	38.38
		60–65	8	4.32
		>70	8	4.32
	Multiple injections	20/15/10	1	0.54
		20/25/35	1	0.54
		25/25/25	5	2.70
		45/45	2	1.08
		80/80/80/80/80/80/80/80	1	0.54
Chemically induced: STZ + prepared rhizome of rehmannia aqueous	One-time injection	30	2	1.08
		53	1	0.54
	Multiple injections	30/30/30/30/30	1	0.54
Chemically induced: STZ + dietary induction + prepared rhizome of rehmannia aqueous	One-time injection	55	1	0.54
Chemically induced: alloxan + STZ	One-time injection	30 + 50	1	0.54

Sixteen papers used alloxan for modeling, mostly in a single intraperitoneal injection, and the most frequently used dose was 50 mg/kg (7, 43.75%); see **Table 4** for details.

**Table 4:** Different injection doses and frequency of alloxan.

Injection method	Dose (mg/kg)	Frequency	Proportions (%)
One-time injection	50	7	43.75
	60	1	6.25
	150	1	6.25
	175	1	6.25
	200	1	6.25
Multiple injections	120/120	4	25
	Unknown	1	6.25

### 3.7 Dietary induction

To further analyze the modeling method of STZ intraperitoneal injection combined with dietary induction, this study counted the specific protocols of dietary induction in the relevant literature. The results showed that irregular feeding with high-sugar and high-fat feeds was the most commonly used dietary induction method (61, 57.01%), in which the time pattern of alternating single-day morning and double-day afternoon feeds was mostly used, and this type of feeding might more effectively mimic the dietary irregularity characteristics of human diabetic patients, as detailed in **Table 5**.

**Table 5:** Method and frequency of dietary induction.

Dietary induction methods	Specific methods	Frequency	Proportions (%)
High-sugar and high-fat feeds	Irregular feeding of high-sugar and high-fat diets (morning feeding on a single day and afternoon feeding on both days)	61	57.01
	High-sugar and high-fat feeds	29	27.10
High-calorie feeds	Irregular feeding of high-calorie diets (morning feeding on a single day and afternoon feeding on both days)	5	5.60
	High-calorie feed feeding	1	0.93
High-fat feeds	High-fat feed feeding	8	7.48
	Irregular feeding on high-fat diets (morning feeding on a single day and afternoon feeding on both days)	1	0.93
Irregular diet	Irregular diet (food given on one day, not given on both days)	1	0.93

### 3.8 Modeling cycle

**Figure 5:**
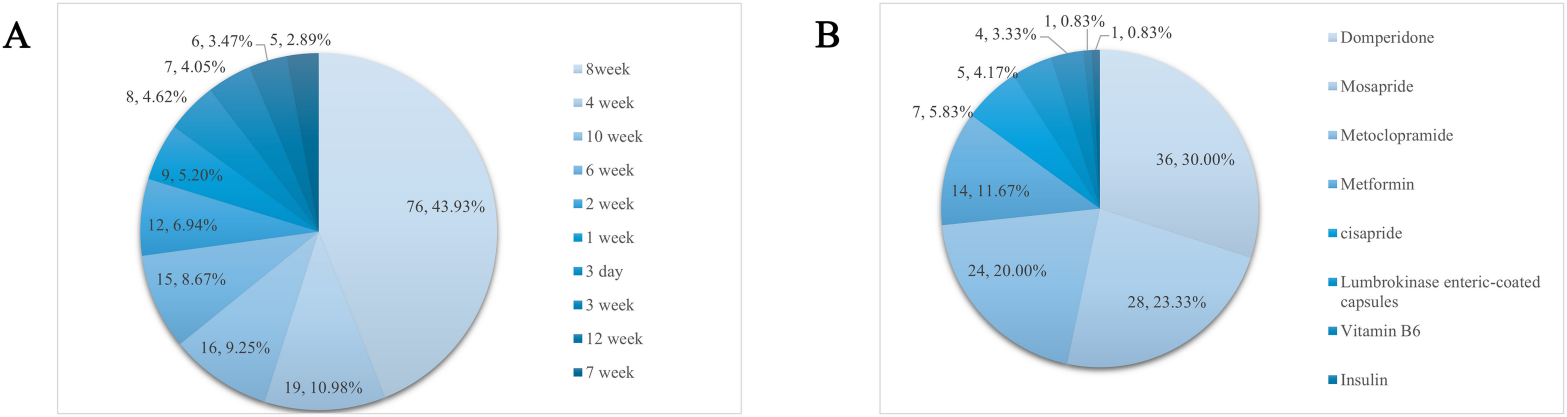
**(A)** Molding cycle and its frequency. **(B)** Frequency distribution of the use of positive controls.

Animal models of DGP are often based on diabetes mellitus and then drug-induced, diet-induced, or natural progression of the disease to the point where the animal develops symptoms related to gastroparesis, so there is variability in the modeling cycle. A total of 207 publications that explicitly recorded modeling cycles were counted, and 32 different cycles were found, of which 10 occurred with a frequency of ≥5. 8 weeks was the most common modeling cycle (76, 36.7%), as shown in **Figure 5A**.

### 3.9 Positive control drug

A total of 110 papers used positive control drugs with a cumulative frequency of 120. Of these, 10 were used in combination, and the most used positive control drug was domperidone (36, 30%), and the rest were, in order, mosapride (28, 23.33%), metoclopramide (24, 20%), and metformin (14, 11.67%). See **Figure 5B** for details.

### 3.10 Mold-forming criteria

Current mold-forming judgments mostly rely on Western medical criteria. The cumulative frequency of modeling criteria was 442 times. Blood glucose, animal general condition, gastric emptying rate, and small intestinal propulsion rate were the main judgment criteria, of which blood glucose was mostly fasting blood glucose or random blood glucose ≥16.7 mmol/L (58, 13.12%). X-ray, gastric blood flow, and *in vitro* smooth muscle experiments were categorized as “other” when they appeared only once. In addition, seven articles only mentioned “gastrointestinal propulsion index” or “gastritis” in the judgment criteria without recording the related methods and data and were also categorized as “other.” For details, see **Table 6**.

**Table 6:** Western medicine modeling criteria and its frequency.

Mold-forming criteria	Frequency	Proportions (%)
Blood glucose (fasting or random blood glucose ≥ 11.1 mmol/L, fasting or random blood glucose ≥ 16.7 mmol/L, etc.)	161	36.43
Gastric emptying rate (test gastric emptying rate using a solution of semi-solid paste, methylene blue, phenol red, and toner)	97	21.95
General conditions (loss of body mass, lack of luster of the animal’s coat, depression, increased water intake and urination, dry and loose stools, etc.)	94	21.27
Small intestinal propulsion rate (test small intestinal propulsion rate using a solution of semi-solid paste, methylene blue, phenol red, and toner)	47	10.63
Other (gastric blood flow, isolated smooth muscle test, x-ray observation of gastric contents and unspecified indicators of gastritis, gastrointestinal propulsion, etc.)	13	3.16
Urine sugar (urine sugar positive)	12	2.71
Gastric myoelectric activity (animals with significantly lower frequency of voluntary gastric contractions compared to the blank group)	6	1.36
Intestinal myoelectric activity (animals with significantly lower frequency of voluntary small intestinal contractions compared to the blank group)	3	0.68
Immunohistochemical observation of nNOS nerve content (significant reduction in the percentage of positively stained area of nNOS neurons in animals)	3	0.68
Electrogastrogram (EEG) (significant difference in EEG amplitude compared to blank control group)	2	0.45
Motilin (significant decrease in animal motilin levels)	2	0.45

### 3.11 Detection indicators

Two hundred eleven pieces of literature were statistically analyzed with a cumulative frequency of about 931 times, and the detection indicators were broadly classified into 11 categories, with a total of 895 detection indicators with a frequency of ≥10, as shown in **Table 7**.

**Table 7:** Frequency distribution of detection indicators.

Detection indicators	Specific indicators	Frequency
Indicators of gastrointestinal motility	Gastric emptying rate, small bowel propulsion rate, electrogastrogram, sinus motility index, gastric electromyographic activity, small bowel electromyographic activity	179
Blood glucose	Animal random blood glucose or fasting blood glucose	135
General condition	Changes in coat gloss, mental status, activity, diet, fecal characteristics, and body mass	132
Western blotting	PI3K, p-PI3K, AKT, p-AKT, nNOS, p-AMPK, NLRP3, Caspase-1, P2X7, Cx43, JAK2, STAT3-mTOR, EGR1, nNOS, Bcl-2, GPR41, GPR43, GPR109A, IGF-1, Bcl-2, Bax, PKA, CaMKII, P-CaMKII, NLRP3, pro-caspase-1, GSDMD, SCF, c-kit, MMP-2, TGF-β3, TGF-β1, TIMP-1, p-NF-κB p65, NF-κB p65, p-IKKβ, IKKβ, p70S6KS6, NRG1, ChAT, α7nAChR, LC3, p62, PGP9.5, GRP78, CHOP, ATF6, and Caspase-12	82
Immunohistochemistry	c-Kit, ERG1, IGF-1, p-PI3K, p-Akt, camp, PKA, CaMKII, P-CaMKII, type I and III collagen in gastric smooth muscle tissue, interstitial cells of Cajal (ICC), PGP9.5, GRP78, CHOP, ATF6, Caspase-12, TMEM16A, ROCK2, nNOS, SYN, SIRT1, Bach1, c-kit, HO-1, CD8, RhoA, MYPT1, p-MYPT1, HIF⁃1α, VEGF, and c-fos, GFAP	73
ELISA	TNF⁃α, IL⁃6, GAS, MTL, GLP-1, PYY, 5-HT, VIP, IL-1β, SP, ACh, NLRP3, IL-18, IGF-1, Ghrelin, CCK, SCF, MOD, SOD, SS, CCK, MOT, GLG, ADP, AMP, ATP, IgA, INS, and IR	71
PCR	Bcl-2, Bax, Caspase-3, JAK2, STAT3, nNOS, ChAT, EGR1, DUSP2, CCL5, GPR41, GPR43, GPR109A, SCF, c-kit, RAGE, PI3K, AKT, p38 MAPK, 5-HT4, PKA, NLRP3, Caspase-1, GSDMD, IL-1β, Nrf2, HO-1, Trx, PI3K-F, PI3K-R, AKT-F, AKT-R, HO-1, NrF2, NOX4, and PGC-1	52
histopathological analysis	Hematoxylin-eosin staining, Masson staining, and Nissl staining were used to observe the pathological changes in gastric or intestinal tissues.	46
Biochemical index	TG, TC, LDL, LDH, MLT, MDA, CAT, ALT, SOD, FINS, IRI, GSH, IgG, and IgA	36
Transmission electron microscopy	Ultrastructure of gastric sinus tissue, ultrastructure and number of ICC, and level of cellular autophagy	27
Immunofluorescence	NRG1, ChAT, α7nAChR, IGF-1R, CHAT, SCF, Ghrelin, gastrictrin, GRP78, LC3, Synapsin-I, and GSDMD-N	14
Insulin resistance	Oral glucose tolerance test (OGTT), glycosylated hemoglobin (HbA1c), insulin, and insulin resistance index (HOMA-IR)	14
Flow cytometry	Observation of apoptosis and proliferation of gastric smooth muscle and other cells, etc.	13
Tunnel staining	Observation of apoptosis, etc.	11
Urine glucose	Positive urine sugar	10

### 3.12 Association rule analysis

**Figure 6:**
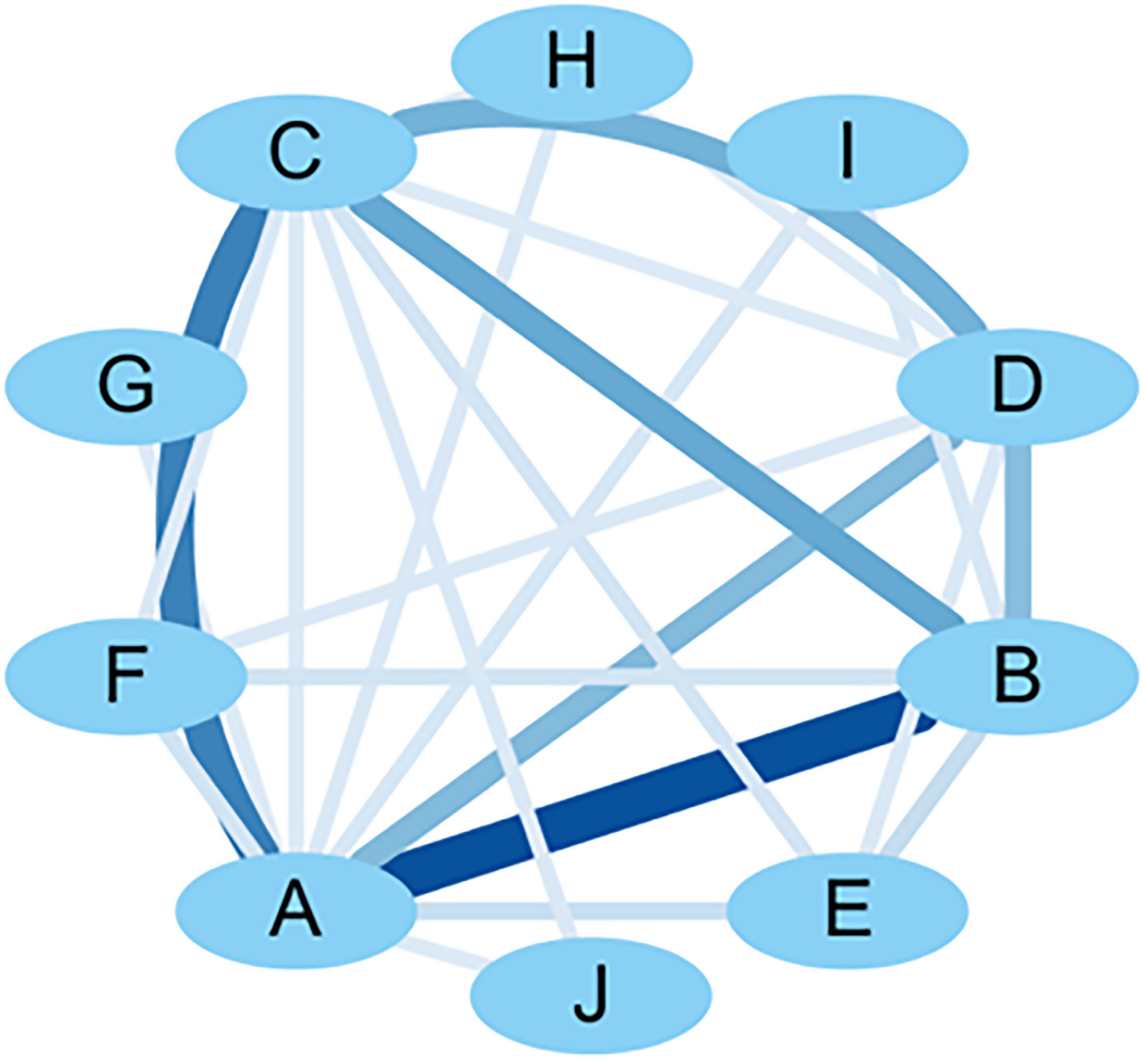
Visualization network diagram of molding criteria correlation analysis. A: blood glucose; B: general condition; C: gastric emptying rate; D: small bowel propulsion rate; E: other small bowel propulsion rate; F: gastric EMG activity; G: electrogastrogram; H: gastrin; I: small intestinal EMG activity; J: OGTT.

SPSS Modeler 18.0 was used to analyze the association rules for the model-forming standard indicators, and the minimum conditional support was set at 10% and the minimum rule confidence was set at 80% to obtain the association network diagram of the detection indicators, and then the association network diagram was visualized and upgraded using Cytoscape 3.7.2. The results showed that the support degree of blood glucose and animal general condition indicators was higher (support degree = 44.08%), indicating that the two are often detected synchronously. Combining the support of the association rule analysis results and the visual network diagram, it can be seen that “A - B - C,” i.e., “blood glucose - general condition - gastric emptying rate,” has a high probability of occurring at the same time, and the three are highly correlated (confidence = 97.62%). The results of random blood glucose or fasting blood glucose, the general condition of the animals (e.g., diet, hair, and body weight), and the gastric emptying rate corroborated with each other and formed an important basis for evaluating the success of DGP modeling. This result validated the modeling assessment criteria of the DGP animal model from multiple dimensions. See **Table 8** and **Figure 6** for details.

**Table 8:** Results of association rule analysis of DGP animal model formation criteria.

Post-term	Pre-term	Instance	Support (%)	Confidence (%)	Gain
Blood glucose	Gastric emptying rate and general condition	42	19.91	97.62	1.28
Blood glucose	Small bowel propulsion rate, gastric emptying rate, and general condition	23	10.90	95.65	1.25
Gastric emptying rate	Small bowel propulsion rate and general condition and Blood glucose	25	11.85	88.00	1.95
Blood glucose	General condition	93	44.08	84.95	1.11
General condition	Small bowel propulsion rate, gastric emptying rate, and blood glucose	26	12.32	84.62	1.92
Blood glucose	Small bowel propulsion rate and general condition	30	14.22	83.33	1.09
Gastric emptying rate	Small bowel propulsion rate and blood glucose	32	15.17	81.25	1.80

## 4 Discussion

Diabetes mellitus and its complications are a highly publicized health problem worldwide. Studies predict that, by 2030, approximately 643 million people (11.3% of the global population) will have diabetes, and this number will continue to rise (20). Global health expenditure on diabetes has increased by 316% in the last 15 years. Gastroparesis is a common consequence of chronically high blood glucose levels in diabetic patients, while gastroparesis can cause changes in the rate of food emptying, leading to fluctuations in blood glucose, again exacerbating the symptoms of gastroparesis (21). In recent years, it has been found that traditional Chinese medicine (TCM) compounds and TCM active ingredients have better effects in improving intestinal mucosal damage, oxidative stress, inflammatory response, and smooth muscle apoptotic response in DGP (22–25). The stable clinical efficacy of TCM on DGP has led to increasing attention to its basic research, so stable and easily reproducible animal models can be the cornerstone of DGP treatment research.

Evaluation by the SYRCLE tool revealed the following bias risk issues in current animal experimental studies on DGP: a. Inadequate description of randomization methods: Most studies only mentioned “randomized grouping” without specifying the specific approach (e.g., random number tables and computer-generated sequences), making it impossible to determine whether the randomization principle was followed. Additionally, random sampling was not explicitly stated in the outcome assessment, potentially introducing measurement bias. b. Lack of blinding implementation: Neither intervention providers nor outcome assessors adopted blinding, which may lead to observer bias and performance bias, thereby overestimating or underestimating the research effect. The combined absence of randomization and blinding may significantly amplify the deviation of intervention effects. c. Insufficient reporting of baseline characteristics: All studies failed to describe animal baseline characteristics in detail (e.g., strain, age, body weight, and health status). Given the generally small sample sizes and large individual variations in animal experiments, missing baseline data reduce comparability between groups, confound intervention effects with baseline differences, and affect the reliability of result inference. d. Omission of experimental environment description: 36.02% of studies did not specify housing conditions (e.g., temperature, humidity, light cycle, and stocking density), which may cause inconsistent experimental conditions and further induce performance bias. e. Data integrity issues: 28.44% of studies reported missing data or reduced sample sizes but did not explain the reasons for animal exclusion or the handling methods nor analyzed the impact of missing data on results. This may introduce selective reporting bias and negatively affect research conclusions.

### 4.1 Modeling animals

Through statistical analysis, it was found that rats with 90% similarity to human genes were mostly used for DGP animal models. Among them, SD rats are predominantly used, and it has been shown that, compared with Wistar rats, they have a lower mortality rate in modeling, probably due to their lower sensitivity to modeling reagents (26). Meanwhile, a few Wistar rats will develop diabetes on their own in the later stages. In addition, both SD rats and Wistar rats have the advantages of low cost, fast reproduction, and easy handling. NOD mice are spontaneous models that can naturally develop into type 1 diabetes mellitus, exhibiting hyperglycemia and insulitis (27). GK rats can also naturally develop into type 2 diabetes mellitus, exhibiting hyperglycemia and insulin resistance. Spontaneous animal models are more compatible with disease progression and mechanisms, but they are long and costly to induce. Therefore, in DGP animal experiments, comprehensive considerations need to be made according to the specific needs of the study and experimental conditions to ensure the reliability of the model and the reproducibility of the results.

Despite the higher prevalence of DGP in human females, male rats are mostly used in animal experiments to avoid the influence of estrogen (28). Nitric oxide synthase (nNOS) activity is closely related to smooth muscle motility, and it has been shown that, in diabetic disease, neural nNOS dimerization and activity are more severely inhibited, and delayed gastric emptying is more pronounced in females (29). Meanwhile, Racine et al. noted that only male mice develop fasting hyperglycemia, fasting hyperinsulinemia, and insulin resistance after induction of a high-fat diet combined with STZ (30), which coincides with the pathophysiology of DGP. Therefore, male animals are more often used in animal testing for DGP because of their stable hormone levels, ease of experimental manipulation, and relative consistency of pathophysiological mechanisms. The use of female animal models may be considered if the effects of gender differences or specific treatments on female animals with DGP are explored.

### 4.2 Modeling substances and methods

DGP animal models are mainly established by chemical inducers, such as STZ or alloxan, and chemical reagent induction can lead to stable hyperglycemia for a short period, which has the characteristics of low cost and simple operation. Alloxan can destroy pancreatic β-cells, but it is less used nowadays because of its lower effectiveness and higher side effects, as well as high mortality rate and risk of spontaneous recovery (31). STZ can bind to the GLUT2 receptor on the membrane of pancreatic β-cells and specifically destroy pancreatic β-cells. However, different injection methods and doses can lead to different structural and cellular changes in animal islets (32). Compared with low doses, high doses can lead to a more rapid progression of islet β-cell loss of function and more severe islet structural destruction. Compared with a single injection, multiple injections of low-dose STZ can reduce the acute toxicity of STZ while ensuring a stable hyperglycemic state and preserving some pancreatic β-cells, but the induction time is longer. Therefore, different doses of STZ can induce different types of DGP, with the high dose (60–65 mg/kg, single injection) mostly used for type 1 DGP, whereas the medium dose (40–55 mg/kg) can mimic type 2 DGP. It has also been suggested by some researchers (33) that type 2 diabetes modeling induced by STZ at an injectable dose of 35 mg/kg may be optimal. Researchers (34) found that low-dose STZ increased the expression of genes related to apoptosis and inflammatory response more than high doses. These differences need to be taken into account in the DGP animal modeling process. STZ-induced models do not show pathophysiology such as insulin resistance, so they are mostly combined with dietary induction in the establishment of animal models of type 2 DGP. In dietary induction, most researchers used high-sugar and high-fat feed irregularly, which can mimic the human disease process of obesity, insulin resistance, and other metabolic disorders. On the one hand, it maps the characteristics of diabetic patients who overeat fat, sweet, and thick flavor, and, on the other hand, it adopts an irregular diet, which makes the animals’ spleen and stomachs rise and fall out of order and cause disease, which is in line with the pathogenesis of diabetes and also combines with the “evidence” of TCM. In addition, the decoction of prepared rhizome of rehmannia (prepared rhizome of rehmannia aqueous) can induce symptoms of abdominal distension by increasing the burden on the spleen and stomach (35), which may lead to delayed gastric emptying by inhibiting the contraction and peristalsis of the gastrointestinal tract smooth muscle (36), but it is different from the delayed gastric emptying induced by hyperglycemia in DGP. Spontaneous models compound the developmental characteristics of human diseases, but the high cost and conditions of reproduction have not yet become the mainstream modeling method. In conclusion, model selection needs to weigh the inducer properties, dose effects, and research objectives.

### 4.3 Modeling cycle

The modeling of DGP is often based on diabetes mellitus, which develops into the appearance of symptoms of gastroparesis, so most experiments feed a period before and after the injection of chemoattractants to promote the development of the disease. However, the time of development of DGP varies, with most researchers opting for 4–10 weeks, with 8 weeks being the hottest choice. It was found that diabetic rats developed gastric hyperdynamia at week 6 (37), and symptoms of gastroparesis were most pronounced at week 8 (38). Some researchers used ultrasound to record the sinus contraction frequency and liquid gastric emptying in rats from 6 to 12 weeks after STZ injection, and the results showed that at weeks 6, 8, 10, and 12, the sinus contraction frequency and gastric emptying rate were significantly lower in the model group compared to the control group.

### 4.4 Positive control drugs

Positive control drugs are the gold standard of scientific research, and their core value is to ensure experimental validity, improve the credibility of results, and assist in decision-making for new drug development. The analysis found that domperidone was the most used positive control drug. As a dopamine receptor antagonist, it significantly shortens the gastric emptying time in the STZ-induced DGP rat model with low blood-brain barrier passage and few central side effects (39). In comparison, the use of metoclopramide is currently limited due to its significant central side effects. Mosapride has a long-term effect of improving gastric motility and can partially reverse gastric smooth muscle dysfunction with long-term use (40). In STZ combined with diet-induced gastroparesis in animals with type 2 diabetes mellitus, metformin can be added to the prokinetic drugs for glycemic control. It is worth emphasizing that interspecific variations in anatomical structures, metabolic pathways, and gene expression can substantially influence drug action mechanisms and therapeutic outcomes. In this study, a rat model was utilized in 88% of the included literature, a result that allows the differences in drugs and mechanisms of different species in DGP animal experiments to be overlooked and fails to discern the relevance of the positive control drug to the species context. Notably, overlooking species-specific contexts may lead to misattributing model-dependent characteristics to universal drug mechanisms. Therefore, clarification of species background is imperative to reduce mechanistic misunderstandings, and there is a need to strengthen the design of species diversity and cross-species mechanistic analyses in future surveys.

### 4.5 Modeling criteria

The DGP animal model is in a hyperglycemic state, so blood glucose is the basic index for judgment, with 16.7 mmol/L or 11.1 mmol/L as the standard line for judgment. Behavioral changes such as abdominal distension, diet, body weight, hair, and stool characteristics are usually combined with the general condition of the animal. However, subjective symptoms such as nausea, abdominal distension, and vomiting cannot be judged, so observing the general condition alone is too subjective and one-sided. Delayed gastric emptying is the core feature of DGP, so the gastric emptying rate is often used to judge the gastrointestinal function of animal models of power. The existing experiments mostly use semi-solid paste, toner, phenol red, methyl orange, methylene, and other pigmented liquids for the power of the judgment of the method. The operation of this type of method is simple and economical, but the rats cannot continue to survive. The delay in gastric emptying is not an inevitable phenomenon, which may lead to errors in the study. The ^13^C breath test is non-invasive, simple, and easy to do. The test is a non-invasive and simple method to detect gastric emptying rate, but the test is long and costly (41). Radionuclide imaging is the “gold standard” for evaluating gastric emptying, which is non-invasive and reproducible, but the equipment is demanding and expensive. Ultrasound can be used to record the contraction amplitude of the gastric sinus and the gastric sinus dynamics index to judge the gastric dynamics of the DGP rat model, but it is susceptible to the interference of the position, intragastric gas, and other factors (42). Some studies have proposed the use of transcutaneous fluorescence spectroscopy to measure gastric emptying rate rapidly and non-invasively (43). Currently, gastrointestinal kinetic indicators are mostly dependent on end-of-life experiments and lack *in vivo* dynamic monitoring tools and cost-effective long-term tracking techniques need to be developed in the future. Comprehensively analyzing the criteria for model formation and the results of correlation analysis, the main indicators for evaluating the success of the DGP animal model include blood glucose, general condition, and the kinetic indicators of gastrointestinal function (e.g., gastric emptying rate and small bowel propulsion rate).

### 4.6 Detection indices

The current detection indexes are mostly based on the criteria for judging the success of the model, plus immunoprotein blotting, immunohistochemistry, immunofluorescence, histopathological analysis, and a variety of PCR to improve the research content. The main research focuses on ICC lesions, gastric smooth muscle lesions, oxidative stress, inflammation, ENS, intestinal homeostasis, etc., which involves signaling pathways such as the Janus kinase 2/signal transducer and activator of transcription 3 (JAK2/STAT3), phosphoinositide 3-kinase/protein kinase B (PI3K/Akt), Kelch-like ECH-associated protein 1/nuclear factor erythroid 2–related factor 2 (Keap1/Nrf2), cyclic adenosine monophosphate/protein kinase A (cAMP/PKA), stem cell factor/tyrosine kinase receptor (SCF/c-kit), and activating transcription factor 6/the C/EBP homologous protein (ATF6/CHOP). The index test was categorized into the following groups: a. JAK2/STAT3 signaling pathways: The JAK2/STAT3 pathway is a critical signaling cascade involved in the inflammatory response. Modulating this pathway can suppress the development of inflammation, alleviate inflammatory damage in DGP rats, and protect the gastric mucosa (44). b. PI3K/Akt/mTOR signaling pathways: PI3K/Akt signaling pathway is a key signaling pathway in DGP, which is closely related to inhibition of apoptosis in ICC (25), activation of autophagy in ICC (45), inhibition of apoptosis in gastric smooth muscle (40), and regulation of oxidative stress (46). In DGP rats induced by STZ alone, activation of the PI3K/Akt pathway may promote smooth muscle apoptosis (47). In DGP rats induced by STZ combined with a high-fat/high-sugar diet, this pathway may contribute to ICC apoptosis (25). c. Keap1/Nrf2 signaling pathways: Nrf2 induces the expression of antioxidant enzymes (48). The Keap1/Nrf2 signaling pathway is a classical signaling pathway against oxidative stress, which can be modulated to inhibit oxidative stress in DGP rats (49). d. cAMP/PKA signaling pathways: In pancreatic β-cells, the cAMP/PKA pathway mediates glucose-stimulated insulin secretion (50). In DGP rats, FoxiangSan treatment significantly reduced hyperglycemia, rebalanced gut microbiota, and upregulated the expression of 5-HT4, cAMP, PKA, and pPKA in gastric sinusoids (10). e. SCF/c-Kit signaling pathways: The SCF/c-Kit pathway is crucial for ICC function, regulating their proliferation, differentiation, and structural integrity, thereby influencing gastric motility (51). f. ATF6/CHOP signaling pathways: The ATF6/CHOP pathway plays a key role in endoplasmic reticulum stress (ERS) regulation. Studies suggest that ERS contributes to gastric smooth muscle dysfunction in DGP rats, and targeting the ATF6/CHOP pathway may inhibit apoptosis and repair smooth muscle damage (25).

## 5 Limitations

However, the following problems still exist in the current DGP animal model: a. Insufficient specificity of the modeling criteria: The existing criteria are overly dependent on blood glucose indicators. Some of the studies were even based on blood glucose values only, which could not distinguish diabetes mellitus from DGP. In addition, there is a lack of objective quantitative criteria for assessing the general state of the animals (e.g., mobility and hair luster). b. Testing methods are invasive and lagging: gastric emptying rate and small intestinal propulsion rate need to be taken at the end of the day, which is difficult to monitor dynamically. Recent studies have attempted to use ^13^C-Spirulina Stable Isotope Gastric Emptying Breath Test (52), and techniques such as ultrasound gastric emptying test and body surface gastric markers may provide non-invasive alternatives (53). c. Insufficient differentiation between type 1 and type 2 DGP models: Most existing animal models simply differentiate between type 1 and type 2 DGP by modeling, but there is a lack of systematic comparative studies between the two in terms of the abnormal pattern of gastric electrical activity, the number of ICCs, and other key pathologic indicators. This leads to a lack of fit between the model and clinical patient characteristics, limiting its translational value. d. Limitations of Chinese medicine evidence type construction: In current animal studies, DGP models are mostly constructed by combining high-sugar and high-fat diets, typically using fixed ratios of high-sugar (such as 20% sucrose) and high-fat (such as 15% lard) feeds as the intervention basis. However, this approach overlooks complex factors in clinical patients’ diets, including protein imbalance, dietary fiber deficiency, emotional stress, and internal injury from overstrain (54). This limitation has led to existing DGP models predominantly focusing on evidence types of Spleen-Stomach Qi Deficiency syndrome, whereas model construction for TCM syndromes such as Liver-Stomach Disharmony and Phlegm-Blood Stasis Obstructing Collaterals remains in a blank state. Additionally, current modeling standards mainly rely on objective Western medical indicators like gastric emptying rate, failing to systematically integrate the evaluation system of TCM’s four diagnostic methods (inspection, auscultation and olfaction, inquiry, and palpation). Future research should prioritize establishing a standardized animal four-diagnosis evaluation system and designing composite intervention protocols based on the principle of “etiological simulation,” such as constructing a Liver-Stomach Disharmony syndrome model through emotional stress combined with a high-sugar diet. e. The methodological quality and experimental reporting quality of animal studies on DGP have problems of varying degrees. In future related experimental studies, researchers should fully recognize the importance of referring to the SYRCLE tool for study design, implementation, and reporting, to improve the credibility of research results and the standardization of reports and to further enhance the clinical translation of research achievements in DGP animal experiments.

## 6 Conclusion

Through data analysis, we found that male SD rats are the preferred animal model for DGP. The modeling methods include a one-time injection of STZ (60–65 mg/kg) to induce type 1 DGP or a one-time injection of STZ (40–55 mg/kg) combined with high-sugar and high-fat chow irregularly fed to induce type 2 DGP. The modeling period was 8 weeks, and model validation criteria were based on blood glucose, general condition, and gastric emptying rate. Domperidone was used as the positive control drug. In addition to model validation criteria, the main detection indexes included histopathological analysis, Western blotting, and immunohistochemistry, which helped improve relevant research. Although there is no universally accepted modeling method or evaluation standard for DGP animal models, this paper systematically analyzed the animal type, gender, modeling methods, validation criteria, positive control drugs, and detection indexes of existing DGP models. The goal is to establish a more economical and reproducible animal model and promote the standardization of DGP animal models.

## Data Availability

The raw data supporting the conclusions of this article will be made available by the authors, without undue reservation.
